# The Relationship Between Aortic Stenosis and the Possibility of Subsequent Macular Diseases: A Nationwide Database Study

**DOI:** 10.3390/diagnostics15060760

**Published:** 2025-03-18

**Authors:** Chia-Yi Lee, Shun-Fa Yang, Elsa Lin-Chin Mai, Jing-Yang Huang, Chao-Bin Yeh, Chao-Kai Chang

**Affiliations:** 1 Institute of Medicine, Chung Shan Medical University, Taichung 40201, Taiwan; 2 Nobel Eye Institute, Taipei 10041, Taiwan; 3 Department of Ophthalmology, Jen-Ai Hospital Dali Branch, Taichung 41265, Taiwan; 4 Department of Medical Research, Chung Shan Medical University Hospital, Taichung 40201, Taiwan; 5 Department of Optometry, MacKay Junior College of Medicine, Nursing, and Management, Taipei 11260, Taiwan; 6 Department of Ophthalmology, Far Eastern Memorial Hospital, Taipei 22060, Taiwan; 7 Department of Emergency Medicine, School of Medicine, Chung Shan Medical University, Taichung 40201, Taiwan; 8 Department of Emergency Medicine, Chung Shan Medical University Hospital, Taichung 40201, Taiwan; 9 Department of Optometry, Yuanpei University of Medical Technology, Hsinchu 30015, Taiwan

**Keywords:** aortic stenosis, age-related macular degeneration, retinal vascular occlusion, epidemiology, TriNetX database

## Abstract

**Objectives**: This study aimed to investigate the possible relationship between aortic stenosis (AS) occupancy and the incidence of subsequent macular diseases. **Methods**: A retrospective cohort study was conducted using the TriNetX database, and participants with AS were enrolled and matched to non-AS participants. A total of 421,860 and 421,860 participants were evenly divided into the AS and non-AS groups, respectively. The major outcomes of the present study include the development of age-related macular degeneration (AMD), retinal vascular occlusion (RVO), epiretinal membrane (ERM), and central serous chorioretinopathy (CSC). Cox proportional hazard regression was utilized for statistical analysis. **Results**: There were 4426 and 3013 AMD events; 7315 and 4753 RVO events; 2780 and 1910 ERM events; and 113 and 64 CSC events in the AS and non-AS groups, respectively. According to the results of Cox proportional hazard regression analysis, the AS group demonstrated significantly higher incidences of all macular diseases, including AMD, RVO, ERM, and CSC, compared to the non-AS group (all *p* < 0.05). The cumulative probabilities of all macular diseases were significantly higher in the AS group than in the non-AS group (all *p* < 0.05). In the sensitivity analysis, the developmental risks of AMD were significantly higher in the AS group than in the non-AS group with all traits. **Conclusions**: This study determined that AS occupancy is related to a higher risk of developing macular diseases, which positively correlated to the disease time of AS.

## 1. Introduction

Aortic stenosis (AS) is a disease that presents with valve fibrosis, valve calcification, and valve structure thickening in the aorta [1,2]. In Western countries, AS prevalence is approximately 5 percent in men and women older than 65 years [3]. Medical interventions can be utilized in mild AS cases, while aortic valve replacement surgery is recommended in patients with severe AS [4,5]. Despite satisfactory survival times after aortic valve replacement [6,7], postoperative AS complications can lead to death [3].

The relationships between AS and several illnesses have been examined in earlier studies [2]. Hypertension is associated with AS formation, and concurrent hypertension in patients with AS can aggregate the symptoms and extent of AS [8]. Regarding blood lipids, hypercholesterolemia can weaken cardiovascular tissues, and dyslipidemia status can alter the risk and treatment outcomes of AS [9]. Furthermore, the risk of diabetes mellitus is significantly higher in people diagnosed with AS [10]. In addition to metabolic syndromes, increased inflammatory conditions and biomarkers have been found in patients with AS [11].

The macular region is located in the central retina, which is a crucial region of the eye that accounts for over half of our visual functions [12]. Age-related macular degeneration (AMD) and retinal vascular occlusion (RVO) are both diseases of the macular region that can contribute to severe visual impairment [13,14]. However, studies that evaluate the potential relationship between the presence of AS and the risk of future macular diseases are lacking in the literature. Because the inflammatory response increases in both AS and macular diseases [11,14,15], a positive relationship between them may exist, which would need verification. Also, AS is frequently associated with the formation of hypertension [16], and this hypertension may contribute to arteriolar remodeling and capillary rarefaction, which would cause the dysfunction of microcirculation [17,18]. After that, the abnormal microcirculation may result in damage to the retinal vasculature and the consequent development of macular disease like RVO [19]. Thus, AS may indeed influence the risk of developing macular disease, which needs a survey.

Therefore, in this study, the authors aimed to investigate and analyze the relationship between AS and each outlined macular disease. The relationship between AS and different macular diseases is analyzed separately.

## 2. Materials and Methods

### 2.1. Data Source

We used TriNetX, which is a global federated health study network that connects multiple electronic medical records (laboratory values, diagnoses, procedures, medications, and genomic information) throughout main healthcare organizations. This project was completed by the US Collaborative Network, which involves 67 healthcare institutions. TriNetX is a platform that de-identifies and restores electronic health record data from different healthcare systems, most of which are large academic medical centers with both inpatient and outpatient departments in several regions in the United States. TriNetX Analytics is equipped with a secure, web-based connection to the electronic health records of subjects from hospitals, primary care, and specialty management providers. Accordingly, the TriNetX database covers diverse geographical regions, age ranges, ethnic populations, income degrees, and insurance classes, involving certain commercial insurance, worker compensation insurance, governmental insurance (Medicare and Medicaid), self-paid, the uninsured, and finally, military and Veterans Affairs insurances. The medical data accessible in that TriNetX database draw upon the International Classification of Diseases, Tenth Revision, Clinical Modification (ICD-10-CM) codes, sex, occupation, age, residence place, socioeconomic level, educational level, hospitalization length if presented, laboratory analysis codes, image analysis codes, the surgical codes, the management codes, and the Anatomical Therapeutic Chemical (ATC) codes of medicine.

### 2.2. Data Selection

**Figure 1:**
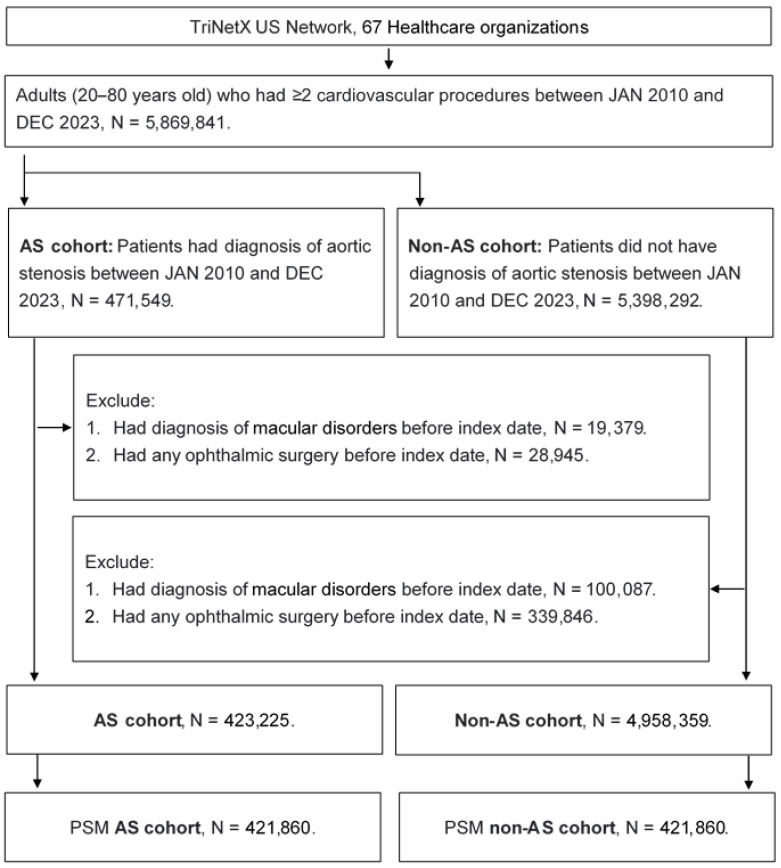
The flowchart of subject selection. AS: aortic stenosis, N: number, PSM: propensity score matching.

A retrospective cohort study was conducted, and participants with the following criteria were selected as patients with AS: (1) AS diagnosis was made according to the ICD-10 CM codes; (2) echocardiogram, electrocardiogram, cardiac catheterization, chest X-ray, or cardiac computerized tomography was performed prior to AS diagnosis; and (3) the AS diagnosis was documented by a cardiologist. The index date in the present study was set to 6 months after the initial AS diagnosis. In addition, the following exclusion criteria were utilized to augment participant homogeneity: (1) age was below 20 years old or above 80 years old; (2) ocular surgery was performed before our index date; and (3) the outcome (displayed in the subsequent section) was found before the index date. Then, one participant with AS was matched to a participant without AS based on the propensity score-matching (PSM) method. The PSM method considers the demography, systemic disorders, and medications in the scoring system and matches different participants with those scores. Of note, non-AS status was assumed rather than proven in the non-AS group. After the PSM method, a total of 421,860 and 421,860 participants were evenly divided into the AS and non-AS groups, respectively. A flowchart of participant selection is shown in Figure 1.

### 2.3. Major Outcome

The major outcomes of the present study are the development of AMD, RVO, epiretinal membrane (ERM), and central serous chorioretinopathy (CSC). The development of AMD was regarded as follows: (1) AMD diagnosis according to the ICD-10 CM codes and (2) the arrangement of a fundoscope exam, optical coherence tomography, or fluorescein angiography according to the procedure code. The development of RVO was regarded as follows: (1) RVO diagnosis according to the ICD-10 CM codes and (2) the performance of a fundoscope exam, optical coherence tomography, or fluorescein angiography according to the procedure code. The development of ERM was regarded as follows: (1) ERM diagnosis according to the ICD-10 CM codes and (2) the performance of a fundoscope exam, optical coherence tomography, or fluorescein angiography according to the procedure code. The development of CSC was regarded as follows: (1) CSC diagnosis according to the ICD-10 CM codes and (2) the performance of a fundoscope exam, optical coherence tomography, or fluorescein angiography according to the procedure code. Only macular diseases observed after the index date would be regarded as the major outcomes in the present study. All participants were traced until macular disease development, they withdrew from all health insurance administrations, or the deadline of TriNetX, 31 December 2023.

### 2.4. Covariate Adjustment

To investigate the relationship between AS and subsequent macular diseases, the influences of the following covariates were considered in the statistical analysis: age, urbanization degree, sex, income amount, hyperlipidemia, hypertension, diabetes, cerebrovascular disease, ischemic heart disease, chronic kidney disease, peripheral vascular disease, chronic pulmonary diseases, HDL, estimated glomerular filtration rate (eGFR), Troponin I, and LDL. The presence of these covariates is in accordance with the corresponding demographic, ICD-10 CM, and laboratory codes. To ensure systemic disease durations were long enough to influence the risk of macular diseases, only diseases that existed for more than two years were considered for the statistical analysis of the present study.

### 2.5. Statistical Analysis

TriNetX calculates the hazard ratios and associated confidence intervals using R’s Survival package v3.2-3. Descriptive analysis was concurrently carried out to illustrate the basic traits of the AS and non-AS groups, and the standard mean difference (SMD) was calculated to determine the difference in each factor between the two groups. An SMD higher than 0.1 was deemed a significant difference in the present study. Then, Cox proportional hazard regression was applied to investigate the incidences of macular diseases between the AS and non-AS groups, and an adjusted hazard ratio (aHR) with a 95% confidence interval (CI) for those incidences of macular diseases was constructed. The demographic data, hypertension, diabetes mellitus, cerebrovascular disease, dyslipidemia, peripheral vascular disease, and laboratory values, including eGFR, Troponin I, LDL, and HDL, were considered in the Cox proportional hazard regression to determine their influence on macular disease development. The Kaplan–Meier curve was plotted, and the cumulative probability of macular disease episodes between the AS and non-AS groups was constructed via the log-rank test. In the sensitivity analysis, the participants were put into different subgroups in accordance with age, sex, race, HDL, and LDL levels. Then, Cox proportional hazard regression was conducted to check the risk of macular diseases in different subgroups. Moreover, the AMD and RVO were divided into dry and wet AMD and branch and central RVO according to the ICD-10 CM codes, respectively, and the correlation between AS and these macular disease subtypes was also analyzed. Statistical significance was regarded as *p* < 0.05, and a *p*-value under 0.001 was exhibited as *p* < 0.001 in the present study.

## 3. Results

The initial traits of the AS and non-AS groups are exhibited in Table 1. The mean age was 65.0 ± 11.8 and 65.5 ± 11.4 years in the AS and non-AS groups, respectively. The difference in age between the two groups did not reveal significant differences. The other demographic data represented insignificant differences between the AS and non-AS groups (all SMD < 0.1). All systemic disease and laboratory data parameters demonstrated no significant difference between the AS and non-AS groups due to the PSM maneuver (all SMD < 0.1) (Table 1).

**Figure 2:**
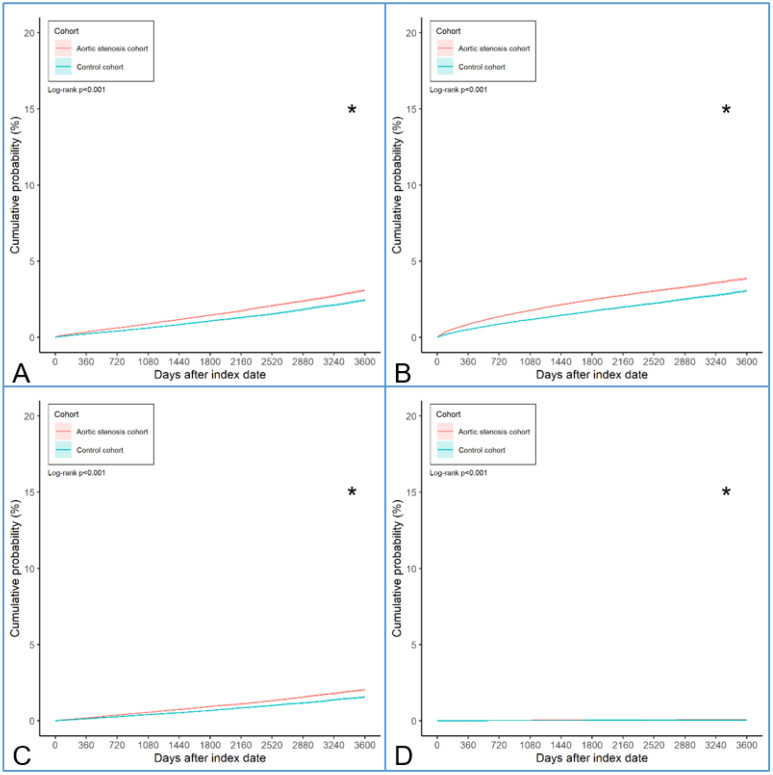
The Kaplan–Meier curve and cumulative incidence of macular diseases between the two cohorts. (**A**) The cumulative incidence of age-related macular degeneration between the two groups. (**B**) The cumulative incidence of retinal vascular occlusion between the two groups. (**C**) The cumulative incidence of epiretinal membrane between the two groups. (**D**) The cumulative incidence of central serous chorioretinopathy between the two groups. * denotes significant differences between groups.

After the entire follow-up interval, there were 4426 and 3013 AMD events; 7315 and 4753 RVO events; 2780 and 1910 ERM events; and 113 and 64 CSC events in the AS and non-AS groups, respectively. In accordance with the Cox proportional hazard regression results, the AS group demonstrated significantly higher incidences of all macular diseases (aHR: 1.400, 95% CI: 1.363–1.438, *p* < 0.001), including AMD (aHR: 1.365, 95% CI: 1.303–1.429, *p* < 0.001), RVO (aHR: 1.437, 95% CI: 1.385–1.490, *p* < 0.001), ERM (aHR: 1.348, 95% CI: 1.272–1.429, *p* < 0.001), and CSC (aHR: 1.637, 95% CI: 1.205–2.224, *p* < 0.001), compared to the non-AS group (Table 2). The cumulative probabilities of macular diseases are shown in Figure 2. The results of the log-rank test represented a higher cumulative probability of AMD, RVO, ERM, and CSC in the AS group than in the non-AS group (all *p* < 0.001) (Figure 2).

**Figure 3:**
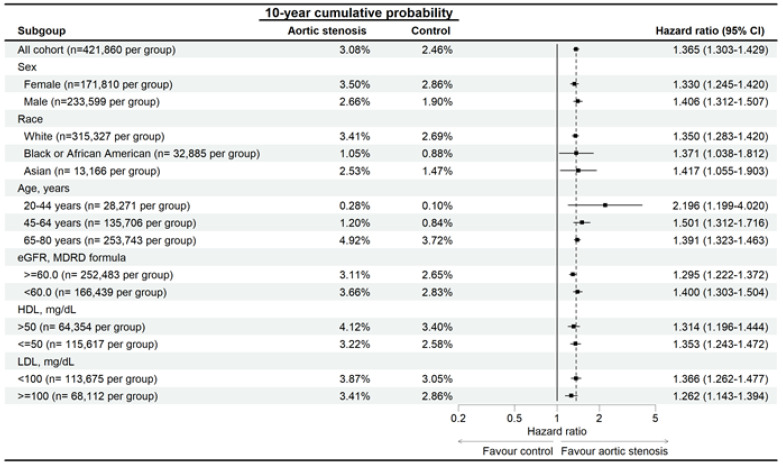
Risk of age-related macular degeneration in patients with aortic stenosis stratified by sex, age, race, HDL, and LDL levels. N: number.

**Figure 4:**
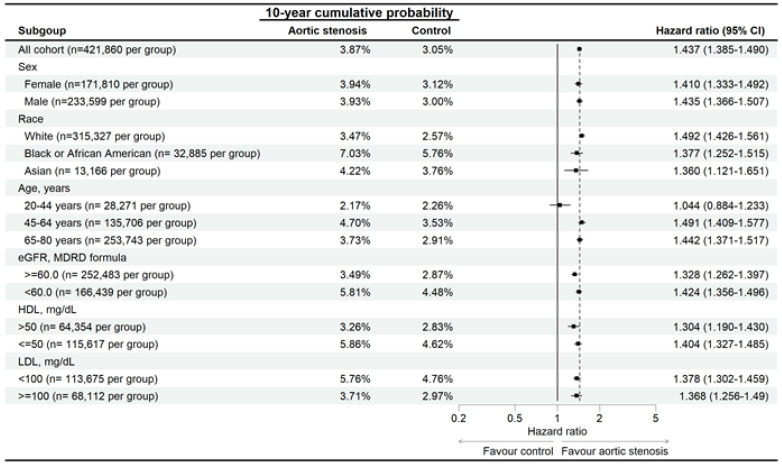
Risk of retinal vascular occlusion in patients with aortic stenosis stratified by sex, age, race, HDL, and LDL levels. N: number.

**Figure 5:**
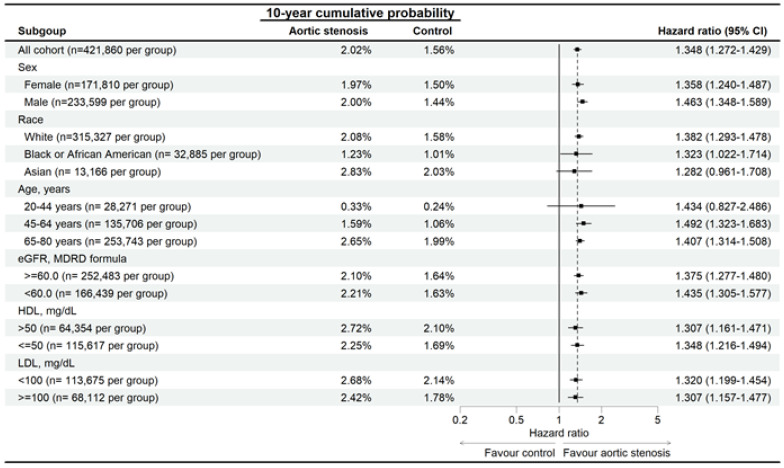
Risk of epiretinal membrane in patients with aortic stenosis stratified by sex, age, race, HDL, and LDL levels. N: number.

**Figure 6:**
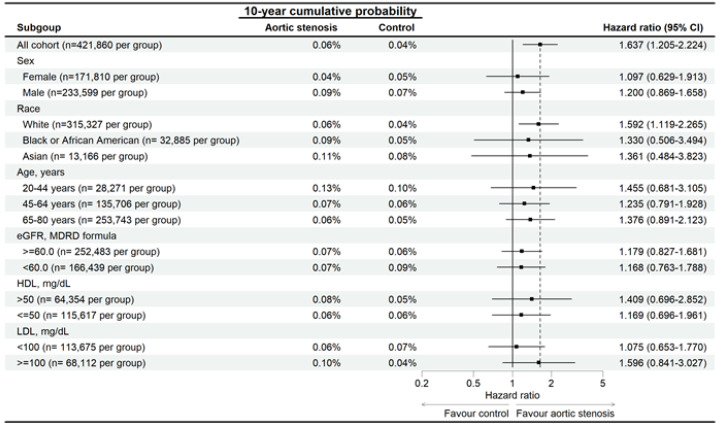
Risk of central serous chorioretinopathy in patients with aortic stenosis stratified by sex, age, race, HDL, and LDL levels. N: number.

In the sensitivity analysis, the developmental risks of AMD were significantly higher in the AS group than the non-AS group for all traits (Figure 3), and the RVO risk was significantly higher in the AS group than in the non-AS group, except for participants aged 20–44 years (Figure 4). The ERM rate was also significantly higher in the AS group than the non-AS group for all traits, except for participants aged 20–44 years and the Asian population (Figure 5). In addition, CSC incidence was significantly higher in the AS group than in the non-AS group only for the whole population and Caucasian populations (Figure 6). Regarding macular disease subtypes, the incidences of dry AMD (aHR: 1.335, 95% CI: 1.273–1.400, *p* < 0.001), wet AMD (aHR: 1.180, 95% CI: 1.070–1.302, *p* < 0.001), branch RVO (aHR: 1.342, 95% CI: 1.184–1.521, *p* < 0.001), and central RVO (aHR: 1.501, 95% CI: 1.366–1.650, *p* < 0.001) were significantly higher in the AS group than the non-AS group.

## 4. Discussion

In summary, the incidence of macular diseases, including AMD, RVO, ERM, and CSC, was significantly higher in participants with AS than those without AS, regardless of the macular disease subtype. Furthermore, the AMD, RVO, ERM, and CSC incidence in the AS group was positively correlated with a longer progression of AS. On the other hand, all AS participants generally demonstrated significantly higher incidences of macular diseases than non-AS participants.

The relationships between AS and certain disorders have been established in previous literature [20]. Metabolic syndromes, defined as dyslipidemia, hypertension, and glucose intolerance, exhibited a positive relationship with AS conditions [21]. Both hypertension and hyperlipidemia are more common in people with pre-existing AS [8,9]. In addition, the possibility of diabetes mellitus was higher in subjects diagnosed with AS, and patients with diabetes and co-existing AS exhibited higher degrees of aortic valve calcification than those without diabetes [10]. Regarding diseases other than metabolic syndromes, neoplasms such as prostate cancer developed more frequently in patients with AS [22]. Inflammation is also a crucial component of AS formation, in which inflammatory activity and lipid accumulation are two prominent mechanisms of the initial occurrence period of AS [23,24]. Moreover, some inflammatory biomarkers were proven to increase with AS development [25]. The levels of both interleukin family and tumor growth factor beta significantly increased in patients with AS [26], and the pathways with bone morphogenic protein and nuclear factor-kappa B significantly increased during AS occurrence [23]. On the other hand, macular diseases are also correlated with elevated inflammation, in which both dry and neovascular AMD presented with increased inflammatory cell aggregation in the choroid [15]. RVO, though from retinal vasculature obstruction [27], also presented with prominent inflammation in the macular region [14]. In addition, pro-inflammatory cytokines were found in ERM tissue [28], and elevated inflammatory biomarkers were observed in the individuals with CSC [29]. Since elevated inflammation was observed in both AS and macular diseases [14,15,25,28,29], we speculated that the presence of AS and elevated inflammatory response may be associated with a higher incidence of later macular diseases. This speculation was justified by the outcomes of the present study.

In the present study, the AS population demonstrated a significantly higher rate of macular disease development compared to the non-AS population. In an earlier publication discussing macular diseases and cardiovascular disorders, coronary heart disorder was found to be associated with a higher risk of RVO [27]. Another study found a significant correlation between early AMD and acute myocardial infarction [30]. Nevertheless, the relationship between AS and macular diseases has not been thoroughly evaluated. To our knowledge, this study is the first to represent the correlation between AS and future macular diseases in the literature, although non-AS status was assumed rather than proven in the non-AS group. Moreover, macular diseases that occurred within 6 months after AS diagnosis were excluded, so the time sequence between AS occurrence and the following macular diseases could be constructed. In addition, we considered several predisposing factors of macular diseases, including age, metabolic syndromes, and serum lipid expressions, in Cox proportional hazard regression [13,31]. It was found that AS may be an independent risk factor for the development of subsequent macular diseases. Vasculature damage was found in some macular diseases, including RVO [31], and vasculature growth was dysregulated in neovascular AMD and CSC [32,33]. Also, the presence of AS could contribute to impaired hemodynamic status and the ischemic condition [24,34]; ischemia is an important predisposing factor for AMD and RVO development, especially for the central RVO [31,32]. Thus, the hemodynamic defect of AS may contribute to the formation of macular diseases like AMD and RVO, in which possible additional therapeutic interventions for the ischemic route may be suggested for AS patients with macular diseases. On the other hand, the presence of AS could cause left ventricular systolic overload, and remodeling and arterial hypertension may also occur in such a condition [35]. Hypertensive status may lead to consecutively high peripheral resistances, and this status could trigger two mechanisms including arteriolar remodeling and capillary rarefaction [36]. Thus, impaired microcirculation is commonly found in patients with hypertension [37]. Due to the existence of impaired microcirculation, the vasculature of the retina would be damaged and RVO may develop under such retinal damage [38]. Except for RVO, defective macular microcirculation was found in AMD via optical coherence tomography angiography [39], and baseline macular microcirculation was lower in eyes with CSC [40]. Overall, the existence of AS may feature poor vasculature status including hypertensive status and dampened microcirculation in the whole body, which is related to macular diseases. The cumulative probabilities of all four macular diseases revealed higher values in the AS group than in the non-AS group. Since the cumulative probability indicates an increased risk of specific complications with the disease period of a specific disorder, people with long-term AS could also present a higher risk of macular disease development than those with short-term AS, for which additional care may be considered.

In the sensitivity analysis, the AS population with specific features revealed generally higher chances of developing AMD, RVO, and ERM than the non-AS population with the same characteristics. Among the specific characteristics analyzed in the present study, some included risk factors for macular disease development, while others did not show a significant correlation with macular diseases [13,31]. These results may further indicate the prominent and general association between AS and subsequent macular diseases. However, young and Asian populations with AS did not demonstrate a higher risk of developing certain macular diseases compared to the same populations without AS. There is a lack of studies that report such findings. Asian ethnicity is not a significant protective factor for macular disease development, except for AMD [13,32]; thus, the findings in the present study may slightly conflict with the earlier literature. A possible explanation for this phenomenon may be the low number of young and Asian cases. In the TriNetx database, both the young and Asian populations only accounted for less than 5 percent of all people and demonstrated a lower number of macular disease cases. Consequently, the low number of young and Asian population may contribute to some statistical bias. A similar situation may also have occurred in the sensitivity analysis of CSC since CSC events were fewer compared to other macular diseases, and this could lead to bias. The aHRs of macular diseases in the young and Asian populations were still higher than one, which might imply that the risk of macular diseases was higher in young and Asian populations with AS. Nevertheless, further research is needed to confirm this hypothesis. Higher incidences of all AMD and RVO subtypes were observed in the AS cohort compared to the non-AS cohort. This phenomenon may indicate that the influence of AS is universal to all macular disease subtypes, and both inflammation and ischemia may account for this universal effect, which needs further validation.

Regarding epidemiology, AS is a prominent cardiovascular defect in the human population, with an incidence of over 3 percent in people more than 75 years old in North America. Over 4 percent of the American population is affected by AS [4,41]. In addition, the AS rate in Chinese patients older than 75 years is high, at 20 percent [42]. Aortic valve replacement has been used for many people with AS in developed countries, but the medical cost is very expensive [43]. Regarding aortic valve replacement, the mortality immediately after aortic valve transplantation is above one percent, despite possible improvements in the overall survival after surgery [4,11]. On the other hand, macular diseases, including AMD and RVO, influence a large proportion of people, and the incidences of both diseases are higher than one percent in the general population [13,31]. In addition, ERM and CSC also develop in a number of people and can affect visual acuity to a high degree [33,44]. Because both AS and macular diseases affect the majority of the population and can lead to severe impairment and high medical costs, any relationship between the two diseases should be represented.

There are certain limitations to the present study. Firstly, the TriNetX database only lists the codes of medical management, while real medical records could not be accessed, except for the values of laboratory examinations. As a result, certain important data, including the sites, degrees, image results, and postoperative conditions of AS; surgical arrangements; AS recurrence; and the site, severity, image results, and therapeutic outcomes of macular diseases could not be evaluated. Secondly, not all participants in the non-AS cohort received an AS-related evaluation, and the image results were not available in the TriNetX database. Consequently, we could not confirm whether a participant was exactly diagnosed with or without AS, and some participants with undiagnosed AS may have been categorized into the non-AS cohort. This limitation would diminish the difference between the two cohorts and AS risk. In addition, the retrospective nature of the present study could diminish participant homogeneity, despite the application of the PSM maneuver to reduce heterogeneity. Moreover, lifestyle factors, such as cigarette smoking, were not included in the analysis because TriNetx does not evaluate these factors, which may alter the incidence of macular diseases like AMD [32]. The health of some AS patients may be extremely poor with long-term bedridden status, and the underlying macular diseases may not have been surveyed in such circumstances. Finally, the age of the study population was high, with more than 60 percent of participants older than 65 years. The comorbidities with advanced age, such as diabetes mellitus, hypertension, dyslipidemia, and chronic kidney disease, may impede the analysis results and reduce the external validity of our findings. Consequently, there are some significant limitations in determining the specific role of AS in the development of the mentioned macular diseases in the present study.

## 5. Conclusions

In conclusion, after considering multiple covariates, AS is determined to be associated with a higher risk of macular diseases involving AMD, RVO, ERM, and CSC. Furthermore, the risks of macular diseases are positively associated with the disease interval of AS. Consequently, periodic ophthalmic examinations may be advised for patients with AS, especially long-term AS. Further large-scale prospective studies that evaluate the relationship between AS and the therapeutic outcomes of macular diseases are needed.

## Figures and Tables

**Table 1:** Baseline traits between the two groups.

Characteristic	AS Cohort	Control Cohort	SMD
N	421,860	421,860	
Age at Index	65.0 ± 11.8	65.5 ± 11.4	0.0418
Sex			
Female	177,396 (42.1%)	175,660 (41.6%)	0.0083
Male	232,157 (55.0%)	232,443 (55.1%)	0.0014
Race			
White	308,671 (73.2%)	312,098 (74.0%)	0.0184
Black or African American	44,917 (10.6%)	42,946 (10.2%)	0.0153
Asian	13,167 (3.1%)	12,602 (3.0%)	0.0078
Comorbidities			
Nicotine dependence	47,011 (11.1%)	55,073 (13.1%)	0.0586
Alcohol-related disorders	15,386 (3.6%)	17,118 (4.1%)	0.0213
Hypertensive diseases	277,688 (65.8%)	280,278 (66.4%)	0.0130
Dyslipidemia	206,752 (49.0%)	208,335 (49.4%)	0.0075
Ischemic heart diseases	167,999 (39.8%)	166,535 (39.5%)	0.0071
Diabetes mellitus	111,224 (26.4%)	109,853 (26.0%)	0.0074
Chronic kidney disease	100,350 (23.8%)	96,621 (22.9%)	0.0209
Peripheral vascular diseases	95,840 (22.7%)	92,775 (22.0%)	0.0174
Chronic pulmonary diseases	76,545 (18.1%)	73,474 (17.4%)	0.0190
Cerebrovascular diseases	65,767 (15.6%)	62,748 (14.9%)	0.0199
Lab data			
eGFR	68.0 ± 30.4	67.4 ± 29.5	0.0190
HDL	46.6 ± 18.4	46.5 ± 18.5	0.0053
LDL	93.4 ± 39.5	97.3 ± 40.4	0.0972
Troponin	1.3 ± 10.6	1.6 ± 15.2	0.0264

AS: aortic stenosis, eGFR: estimated glomerular filtration rate, N: number, SMD: standard mean difference.

**Table 2:** Main outcomes between the aortic stenosis and control cohorts.

Study Event	N	Cumulative Probability				Hazard Ratio (95% CI)	*p* Value
		1 Year	3 Years	5 Years	10 Years		
All macular disorders							
AS (n = 421,860)	13,455	1.33%	3.01%	4.50%	8.05%	1.400 (1.363–1.438)	<0.001 *
Control (n = 421,860)	8994	0.82%	2.04%	3.22%	6.43%	Reference	
AMD							
AS (n = 421,860)	4426	0.34%	0.88%	1.45%	3.08%	1.365 (1.303–1.429)	<0.001 *
Control (n = 421,860)	3013	0.22%	0.61%	1.07%	2.46%	Reference	
RVO							
AS (n = 421,860)	7315	0.85%	1.76%	2.46%	3.87%	1.437 (1.385–1.490)	<0.001 *
Control (n = 421,860)	4753	0.51%	1.16%	1.71%	3.05%	Reference	
ERM							
AS (n = 421,860)	2780	0.18%	0.55%	0.93%	2.02%	1.348 (1.272–1.429)	<0.001 *
Control (n = 421,860)	1910	0.12%	0.40%	0.66%	1.56%	Reference	
CSC							
AS (n = 421,860)	113	0.01%	0.02%	0.04%	0.06%	1.637 (1.205–2.224)	<0.001 *
Control (n = 421,860)	64	0.01%	0.02%	0.03%	0.04%	Reference	

aHR: adjusted hazard ratio, AMD: age-related macular degeneration, AS: aortic stenosis, CI: confidence interval, CSC: central serous chorioretinopathy, ERM: epiretinal membrane, N: number, RVO retinal vascular occlusion. * denotes significant differences between groups.

## Data Availability

The original data used in the current study are not available due to the policy restrictions of the TriNetX database.

## Abbreviations

The following abbreviations are used in this manuscript:

aHR	adjusted hazard ratio
AMD	age-related macular degeneration
AS	aortic stenosis
CI	confidence interval
CSC	central serous chorioretinopathy
eGFR	estimated glomerular filtration rate
ERM	epiretinal membrane
ICD-10	International Classification of Diseases Tenth Revision
N	number
PSM	propensity score matching
RVO	retinal vascular occlusion
SMD	standard mean difference
